# Dietary Diversity and Dietary Quality: Associations With Body Mass Index Change in Diverse Adults With Age

**DOI:** 10.1093/geroni/igaa057.771

**Published:** 2020-12-16

**Authors:** Marie Kuczmarski, Rita Rawal, May Beydoun, Nancy Cotugna, Benjamin Brewer, Virginia Hughes, Alan Zonderman, Michele Evans

**Affiliations:** 1 University of Delaware, Newark, Delaware, United States; 2 National Institute on Aging, Bethesda, Maryland, United States

## Abstract

Dietary Diversity (DD), a variety element of a healthful diet, can be measured by count, evenness and dissimilarity scores. This study explored the associations of DD, and dietary quality measured by Dietary Approaches to Stop Hypertension (DASH) score, with annualized Body Mass Index (BMI) change in a diverse sample. Participants, 1,104 African American (57.9%) and White (42.1%) adults, were from the longitudinal Healthy Aging in Neighborhoods of Diversity across the Life Span study. Mean±SE age at baseline was 48.3±0.20 years. The DD and DASH scores were calculated using four 24-hr recalls from baseline (2004-2009) and 1st follow-up wave (2009-2013). Count was based on consumption of ≥50% of an equivalent from 21 food groups. Evenness was derived using the Berry-Index adjusted by the food’s health value; dissimilarity, by Mahalanobis Distance. The DASH score was computed using the Mellen formula. BMI was calculated from measured weight and height; change in BMI from 1st to 2nd follow-up waves (2009-2017). Linear regression results are expressed as β-coefficients □ standard error of means (β□SE). After adjusting for energy, age, sex, race, poverty status, education, and smoking, of the three DD measures, only mean count was associated with annualized change in BMI (8.166±3.575, p=0.023). Mean DASH score was inversely associated with BMI change (-6.599±2.690, p=0.014). Age and smoking were the only other significant predictors (-1.137±2.938, p<0.001), (-1.169±5.472, p=0.033), respectively. These findings provide evidence that high quality is associated with a decrease in BMI with age while high count scores are associated with a rise in BMI.

